# Development, nutritional profiling, physicochemical properties, computational protein-polyphenol interactions, and sensory properties of high-energy Medjool date bars

**DOI:** 10.3389/fnut.2026.1813284

**Published:** 2026-05-11

**Authors:** Ahmed H. Bahloul, Galal A. Ghazal, Mahmoud H. Mahmoud, Hani A. Alfheeaid, Thamer Aljutaily, Raed Alayouni, Hend F. Alharbi, Hassan Barakat

**Affiliations:** 1 Food Technology Department, Faculty of Agriculture, Benha University, Moshtohor, Egypt; 2 Department of Food Science and Human Nutrition, College of Agriculture and Food, Qassim University, Buraydah, Saudi Arabia

**Keywords:** bioactive compounds, energy value, food supply, high-energy snacks, *Phoenix dactylifera*, rheological behavior

## Abstract

**Background:**

Health-promoting foods that support healthy aging are attracting growing interest, and Medjool dates, energy-dense and rich in bioactives, remain underutilized in protein-based functional snacks.

**Objectives:**

The current study aims to develop and assess high-protein energy bars made with Medjool date paste, focusing on their nutritional, phytochemical, stability, and physicochemical properties.

**Methods:**

Three Medjool date–based protein bars (F1–F3) were formulated with increasing Medjool paste (45–55%) and decreasing oats (10–0%), alongside constant milk protein concentrate–whey protein isolate (20%) and complementary mix (25%), and were characterized for composition, phytochemicals and antioxidant activity, protein–polyphenol interactions (docking), microstructure, thermal behavior, color, texture, and sensory attributes.

**Results:**

Proximate composition analysis demonstrated a high nutritional profile of the fortified date bars. Moisture ranged from 11.81–13.64%, protein 22.98–24.38%, and available carbohydrates 53.27–57.01%. Fat content was 11.20–12.25%, with dietary fiber at 5.84–6.91% and ash at 2.96–3.18%. Total, reducing, and non-reducing sugars were 33.22–41.18%, 31.06–38.86%, and 2.05–2.20%. The resulting energy value of the nutritional date bars remained consistent across formulations (420.76–422.30 kcal per 100 g), indicating overall nutrient density. The antioxidant evaluation revealed total phenolic contents ranging from 562.48 to 619.26 mg GAE per 100 g, and total flavonoid concentrations from 383.42 to 395.43 mg QE per 100 g, indicating stable radical-scavenging performance across all treatments. Molecular docking revealed that epicatechin binds specifically to *β*-lactoglobulin via hydrophobic and *π*-π stacking interactions, and to sodium caseinate via ionic and H-bond interactions. Molecular docking suggested specific binding between epicatechin and *β*-lactoglobulin/sodium caseinate via hydrophobic and hydrogen-bond interactions, indicating potential stabilization of polyphenols in the protein-enriched matrix. The instrumental color assessment showed a uniform brownish-yellow hue, whereas sensory testing yielded consistently high acceptability ratings (86.91–87.82) with no significant variation among the developed formulations. Texture profile analysis showed formulation-specific texture in MEBs: F2 was hardest (199 N), F1 was most cohesive/adhesive (0.69/−0.45), and F3 was springiest/chewiest (0.91/93), optimizing sensory appeal.

**Conclusion:**

The formulated Medjool energy bars were successfully developed, showing a strong nutritional profile, high consumer acceptance, and promise as scalable, stable, nutrient-dense products for athletes and health-conscious consumers.

## 1 Introduction

Foods that promote human health and longevity are gaining increasing attention as alternatives or complements to dietary systems. Date palm *(Phoenix dactylifera L.)* fruits, seeds, and pollen have long served as integral components of traditional diets and medicinal systems and exhibit diverse bioactivities, including antioxidant, anti-inflammatory, antihyperlipidemic, antihypertensive, antidiabetic, antitumor, hepatoprotective, antibacterial, and antiviral effects. Variability in genotype and growing conditions contributes to substantial differences in their nutrient and phytochemical composition, particularly in phenolics, carotenoids, phytosterols, and oxylipins. These bioactive compounds remain underutilized in the food sector despite their potential to enhance the sensory, nutritional, and functional properties of a wide range of products. Owing to their high dietary density, date fruits and their co-products offer promising opportunities for the development of functional foods. Previous review has integrated current evidence on their nutrient composition, biofunctional properties, and factors influencing their abundance and bioactivity (1).

High-energy nutritional bars are increasingly formulated as compact, nutrient-dense food systems designed to meet the elevated metabolic and dietary demands of athletes, shift workers, and other individuals with heightened energy requirements (2). In 2024, the global nutritional bar market was valued at approximately USD 7.4 billion and is projected to reach USD 13.2 billion by 2034, reflecting a compound annual growth rate of 6.1% (3). This expansion is attributed to increasing global health awareness and the growing demand for convenient, nutrient-dense dietary options. Moreover, the functional food sector is projected to reach USD 441.66 billion by 2028, with nutritional bars, particularly protein-enhanced, energy-focused, meal-replacement, and specialty formulations (e.g., gluten-free, organic, and plant-based), representing a significant contributing segment (4). Nutrition bars are widely consumed functional snacks among diverse groups, including young individuals, athletes, vegans, and those adhering to specialized dietary regimens (5). It contains proteins, carbohydrates, fats, vitamins, and minerals (6) and plays multiple functional roles, such as boosting energy (7).

Dates are incorporated into a wide range of food products, including energy bars, which have become increasingly popular (8). Some previous studies have used raw materials such as Amaranth, quinoa, roasted peanuts, raisins, dried fig, and honey (9). Nuts (almond, pistachio, cashew, and walnut), hulled pumpkin seeds, hulled sunflower seeds, chia seeds, and other ingredients were used to develop date bars (3). Medjool dates are a nutrient-dense fruit offering a variety of health benefits, making them a valuable addition to a healthy diet. They are rich in carbohydrates, primarily glucose and fructose, and provide essential nutrients, including vitamin B, dietary fiber, and minerals. Additionally, they contain phenolics, carotenoids, and antioxidants, which contribute to their health-promoting properties (10). They are beneficial for athletic performance due to their high energy content and ability to replenish glycogen stores (11). Despite their high sugar content, Medjool dates have a low glycemic index and do not adversely affect serum glucose or lipid levels, making them suitable for consumption without increasing the risk of chronic diseases. Consuming Medjool dates does not significantly affect serum glucose or lipid levels and may even reduce triacylglycerol levels by 8%. The pilot study investigated the effects of Medjool date consumption on serum glucose, lipids, and oxidative stress in healthy subjects, revealing that both date varieties did not significantly affect body mass index or serum cholesterol levels, while fasting serum glucose and triacylglycerol levels remained stable or decreased after consumption (12).

The chemical composition of Medjool dates (*Phoenix dactylifera* L. family Arecaceae) reveals a rich nutritional profile, characterized by significant levels of sugars, minerals, and phenolic compounds (13). Medjool is a well-known cultivar and one of the highest-quality dates on the market. The large size, soft flesh, and attractive appearance have made it popular among growers and consumers (14). Medjool dates contain significant levels of phenolic compounds, such as gallic acid and flavonoids, which contribute to their antioxidant potential and support the successful development of functional MEBs with an excellent nutritional profile and strong panelist acceptance (15, 16).

Medjool dates were selected as the main ingredient based on evidence showing their enhanced mineral bioavailability and greater phytochemical content compared to other commercial date varieties. The underlying mechanisms of protein-polyphenol interactions in energy bars remain largely unexplored. Prior studies have examined neither the thermodynamic interactions between milk proteins and date-derived bioactives nor applied comprehensive analytical techniques, including Scanning Electron Microscopy (SEM), Differential Scanning Calorimetry (DSC), colorimetry, and texture profiling, to assess how protein addition impacts product functionality and stability. As a result, connections between protein-bioactive complexation, nutrient accessibility, and overall product quality are poorly defined. However, analogous dairy-tea, dairy-cocoa, and dairy-fruit beverage systems, binding of phenolics to casein and *β*-lactoglobulin has been shown to modify phenolic stability. *In vitro* bioaccessibility data are available, but comparable data are not yet available for date bar matrices, which is why the present study employs molecular docking as a first step to explore these potential protein-phenolic interactions (17–20). This research fills these gaps by systematically analyzing such interactions in Medjool date formulations enriched with milk and plant proteins, thus clarifying how strategic protein addition modifies physicochemical attributes and maintains bioactive integrity in date snack systems. Therefore, this investigation aimed to create an innovative high-nutrition bar using Medjool date paste. Three distinct formulations were developed by combining Medjool date paste with milk protein, whey protein isolate, oats, almonds, peanut butter, date syrup, sesame seeds, coconut powder, buffalo ghee, wheat bran, and salt. The resulting MEBs underwent a comprehensive evaluation of their proximate composition, mineral profile, amino acid content, phytochemical makeup, textural attributes, physical properties, protein-polyphenol interactions, and sensory qualities to determine their viability as functional foods. Unlike previous studies on date-based or protein-enriched bars, we hypothesized that incorporating milk protein concentrate and whey protein isolate into Medjool energy bars would (i) enhance protein quality and mineral density, (ii) stabilize key polyphenols through specific protein–polyphenol interactions characterized by molecular docking, and (iii) maintain desirable physicochemical and sensory properties suitable for functional high-energy snacks.

## 2 Materials and methods

### 2.1 Ingredients

Medjool date paste, oats, almonds, peanut butter, and date syrup were procured from Abu Auf Factory (https://www.abuauf.com), March 2025. Milk protein concentrates and whey protein isolate powders were obtained from MYPROTEIN Co., Manchester, UK (www.myprotein.com), January 2025. Sesame seeds, coconut powder, buffalo ghee, and wheat bran were sourced from local markets in Egypt. At the same time, salt was purchased from The Egyptian Minerals and Salts Company at Fayoum (EMISAL), March 2025.

### 2.2 Formulation of MEBs

The MEBs were prepared following a modified protocol adapted from Ibrahim et al. (21). Protein was fixed at 20 g 100 g^−1^ using a 1:1 blend of milk protein concentrates and whey protein isolate, as preliminary trials showed this level met ≥20% (w/w) high-protein bar specifications while maintaining workable dough texture; higher protein caused crumbliness, whereas lower levels failed to achieve the desired protein enrichment. Raw almonds were first ground in a Moulinex Odacio Food Processor (FP7371, Moulinex, France) at speed 3 for 30 s. The dry blend comprising sesame seeds, ground almonds, oat flour, milk protein concentrate, whey protein isolate, wheat bran, and salt was roasted in a convection oven at 200 °C for 5 min with constant stirring, after which coconut powder was incorporated and roasting continued for an additional 3 min at the same temperature. Concurrently, wet components (date paste, buffalo ghee, date syrup, and peanut butter) were heated separately at 120 °C for 5 min to lower viscosity and facilitate blending.

The MEBs were formulated as specified in Table 1. The pre-heated dry and wet components were combined in a dough mixer and blended until a uniform consistency was achieved. The resulting dough was portioned to precise weights using a calibrated balance, then manually formed into bars of standardized dimensions. Finished MEBs were promptly stored under refrigeration at 4 ± 1 °C in sealed containers to minimize between-batch moisture variability. During processing, key control parameters, including temperature, mixing duration, and ingredient homogeneity, were rigorously monitored to maintain reproducibility and quality standards.

**Table 1:** Edible ingredients of formulated MEBs (g 100 g^−1^).

Ingredients	MEBs formulas g 100 g^−1^		
	F1	F2	F3
Mejdool paste	45	50	55
Milk protein concentrate, whey protein isolate (1:1)	20	20	20
Whole oat	10	5	0
Complementary mix*	25	25	25

*The complementary mix contains: 4.5 g Medjool date syrup, 4 g sesame seeds, 4 g ghee, 3 g coconut powder, 2 g wheat bran, 2 g peanut butter, and 0.5 g edible salt.

### 2.3 Chemical composition and minerals content of MEBs

The formulated MEBs were analyzed for proximate composition, including moisture, crude protein, lipids, ash, dietary fiber, and available carbohydrates, along with caloric value, following standard AOAC protocols (2012). Total, reducing, and non-reducing sugars were quantified via the Lane-Eynon volumetric titration (method 935.64) as outlined in AOAC (22). Sodium and potassium were determined by flame photometry using a Jenway flame photometer PFP7 instrument (Cole-Parmer Jenway, Staffordshire, UK) per method 956.01. In contrast, calcium, magnesium, iron, copper, manganese, and zinc levels were assessed by atomic absorption spectroscopy (PerkinElmer 2,380 AAS; PerkinElmer, Waltham, MA, USA) according to AOAC 968.08 (2012). Phosphorus content was measured colorimetrically following the procedure detailed by Borah et al. (23). Mineral contents are expressed on a dry weight basis.

### 2.4 Determination of amino acids profile in MEBs

The amino acid profiles of the experimental samples were determined using the Pico-Tag HPLC (Waters Corporation, Milford, MA, USA) method as detailed by Cohen (24), with minor modifications. Samples were hydrolyzed in 6 N HCl at 110 °C for 24 h under nitrogen; sulfur amino acids were oxidized with performic acid prior to hydrolysis. Post-hydrolysis, residues were reconstituted in sodium citrate buffer (pH 2.2), derivatized with phenylisothiocyanate (PITC) to form PTC-amino acids, and separated on a Pico-Tag C18 column (3.9 × 150 mm, Waters) at 38 °C using a sodium acetate (pH 6.4)–acetonitrile:water gradient (1.0 mL min^−1^), with UV detection at 254 nm. Amino acids were identified and quantified via external standards and calibration curves; analyses were in duplicates and expressed as g g^−1^ nitrogen.

### 2.5 Determination of phytochemical compounds in MEBs

Total phenolic content (TPC) in the MEBs was quantified using the Folin–Ciocalteu assay, with results expressed as mg gallic acid equivalents per 100 g dry weight (mg GAE 100 g^−1^ dw) following the protocol of Bettaieb et al. (25) using the equation (y = 0.0201x + 0.0538), R^2^ = 0.97. Total carotenoid (TC) content was measured colorimetrically according to a modified protocol (26). Antioxidant capacity, assessed via DPPH radical scavenging activity (DPPH-RSA) with results reported as micromoles of Trolox equivalents per gram dry weight (μmol TE g^−1^), was examined (27). Radical scavenging activity (ABTS-RSA) against ABTS radicals was measured using the method described by Almundarij et al. (28). The DPPH-RSA and ABTS-RSA were calculated using the equation (y = 968.96x + 15.605), R^2^ = 0.92. Additionally, total flavonoids (TF) and total flavonols (TFL) contents were determined using methods by Barakat and Almundarij (29) and Kumaran and Karunakaran (30), respectively, with values expressed as milligrams of quercetin equivalents per gram (mg QE g^−1^). The TF and TFL were calculated using the equation (y = 0.1099x + 0.1718), R^2^ = 0.89.

### 2.6 Determination of phenolic and flavonoid compounds in MEBs

Phenolics and flavonoids were separated on an Alliance e2695 HPLC system (Waters Corporation, Milford, MA, USA) equipped with a 2,998 photodiode-array (PDA) detector (190–800 nm wavelength range; 1.2 nm optical resolution; up to 80 Hz sampling; analytical flow cell 10 mm pathlength, 8.4 μL). Detection was set to scan 200–600 nm with chromatograms extracted at compound-specific λmax as applicable. The stationary phase was a C18 column (250 × 4.6 mm, 5 μm; Agilent Technologies, Santa Clara, CA, USA) (31). Solvent A consisted of 5% (v/v) formic acid in water, paired with methanol as solvent B. The elution followed this gradient: from 0 to 10 min, B increased from 5 to 20% and held steady for 5 min more; from 15 to 30 min, B rose from 20 to 25% before holding again for 5 min; from 35 to 40 min, B climbed to 33%; then from 40 to 42 min, it dropped back to 5% with a final 5-min hold. The injected samples (10 μL), flow rate was (1 mL min^−1^), column temperature (30 °C), and the absorbance was monitored over 200–600 nm. The limit of detection (LOD) was < 0.1 μg mL^−1^, the limit of quantification (LOQ) was 0.5–2 μg mL^−1^, and R^2^ = 0.891.

### 2.7 Interactions of polyphenol and protein in MEBs matrix

Molecular docking studies examined the binding interactions between principal milk proteins (*β*-lactoglobulin and sodium caseinate) and epicatechin, a predominant bioactive in energy bar formulations. Three-dimensional structures of β-lactoglobulin (PDB ID: 3NPO) and a representative casein micelle model were obtained from the Protein Data Bank in the RCSB. At the same time, the geometries of epicatechin and caffeic acid were retrieved from PubChem. Simulations were performed using Discovery Studio software, with crystal structures prepared by removing waters, adding polar hydrogens, and applying Gasteiger charges. The workflow encompassed protein preparation, ligand optimization, active site identification, and detailed interaction profiling, including binding energy calculations, 3D visualizations, 2D interaction maps, surface analyses, and PLIP characterization, to reveal molecular mechanisms of protein-polyphenol complexation within date bar matrices. All runs were triplicated for reliability. Indeed, epicatechin was chosen because it was among the predominant phenolics quantified by HPLC and is a well-studied flavan-3-ol with established interactions with milk proteins; note that caffeic acid was also modeled, but epicatechin is highlighted due to its higher levels and relevance to antioxidant function.

### 2.8 Visual instrumental color measurements of MEBs

Color measurements for each sample were performed using a Chromameter (ColorFlex, Reston, VA, USA) on the CIELAB scale (L*, a*, b*) with standard white, green, and black calibration tiles. Hue angle (H°), chroma (C*), and browning index (BI) were subsequently calculated following the method of Lavelli et al. (32). The color differences (ΔE) were calculated using the CIE (ΔE) formula, which quantifies perceptual differences in CIELAB color space between the sample (L*₂, a*₂, b*₂) and reference white tile (L*₁, a*₁, b*₁) as follows:

$$\mathrm{\Delta E}=\sqrt{{\left(L^{*}2-L^{*}1\right)}^{2}+{\left(a^{*}2-a^{*}1\right)}^{2}+{\left(b^{*}2-b^{*}1\right)}^{2}}$$

### 2.9 SEM analysis of MEBs

Medjool energy bar (MEB) samples were subjected to detailed microstructural examination using SEM and digital image analysis in Origin 2025. Specimens were prepared according to standard protocols for food matrices, involving precise cross-sectioning to reveal representative internal surfaces (SU8010, Hitachi Co., Ltd., Tokyo, Japan), followed by conventional SEM techniques, such as critical-point drying or freeze-drying, to eliminate moisture while preserving native architecture for electron-beam imaging. High-resolution SEM micrographs capture surface topography through electron-sample interactions, offering magnifications from 10 × to over 500,000 × with superior depth-of-field control and three-dimensional detail beyond the limits of optical microscopy-ideal for characterizing food microstructures. Post-imaging, micrographs underwent rigorous processing in Origin 2025, encompassing grayscale conversion, binary thresholding for segmentation, and quantitative assessment of surface features. This workflow yielded precise metrics on particle size distribution, porosity, defect profiles, and homogeneity, enabling robust inter-sample comparisons and statistical validation of structural attributes, including crack dimensions, pore morphology, and surface uniformity (33).

### 2.10 DSC analysis of MEBs

DSC analysis was conducted on three homogenized Medjool energy bar (MEB) samples, with 5–10 mg portions hermetically sealed in aluminum pans (DSC; VP-Capillary-DSC Q20, Thermal Analysis Corp., DE, USA); empty sealed pans served as references. The instrument was pre-calibrated using indium standards. Samples were held isothermally at 25 °C for 2 min, then heated to 200 °C at 10 °C/min under nitrogen flow (50 mL/min). Thermal profiles captured heat flow data to determine onset/peak temperatures and enthalpy values. Duplicate runs yielded consistent results, and DSC parameters were further processed in OriginPro 2025 (34).

### 2.11 Texture analysis measurements of MEBs

Texture profile analysis (TPA) of all samples was performed using a T. A. XT Plus texture analyzer (Stable Micro Systems, Godalming, Surrey, UK) fitted with a 50-kg load cell and 75-mm aluminum cylindrical probe (P/75). Uniform sample cubes (20 × 20 × 20 mm^3^) were prepared with a sharp blade, equilibrated at 25 °C for 2 h, and centrally positioned on the test platform. A double-compression cycle was executed at pre−/post-test speeds of 2.0 mm/s, test speed of 1.0 mm/s, 50% strain target (trigger force: 5 g; inter-cycle pause: 5 s). Force-time curves generated via Exponent software (v6.1) yielded key parameters: hardness (maximum first-bite force), cohesiveness (area ratio A2/A1), springiness (recovery distance), chewiness (hardness × cohesiveness × springiness), and adhesiveness (negative work area). Triplicate measurements per formulation were averaged and reported as mean ± SD (35).

### 2.12 Sensory evaluation of MEBs

Sensory evaluation of the formulated date bars was conducted with 12 trained panelists from Benha University’s Faculty of Agriculture. A composite scoring approach was used to rate appearance, color, aroma, taste, and texture, and overall acceptability was derived from the combined attribute scores to reflect general consumer preference. These assessments provide valuable information for product optimization, quality assurance, and targeted marketing applications (36). During sensory evaluation, samples were presented in randomized order and coded with three-digit blinding codes, panelists were blinded to formulation identity, attributes were evaluated using a [e.g., 20-point scoring scale]; overall acceptability was computed as the sum of attribute scores, and sensory procedures were approved by our Ethics Committee (Approval No REC-FOABU 8/00029), and informed consent was obtained from all panelists.

### 2.13 Statistical analysis

Statistical analyses were conducted using SPSS software (Version 27.0 for Windows; IBM Corp., Armonk, NY, USA). Data were checked for normality (Shapiro–Wilk test) and homogeneity of variances (Levene’s test) before one-way ANOVA. One-way ANOVA with Tukey’s post-hoc testing determined significant differences, where *p* < 0.05 was deemed statistically significant following Steel (37).

## 3 Results and discussion

### 3.1 Proximate chemical analysis of formulated MEBs

**Figure 1:**
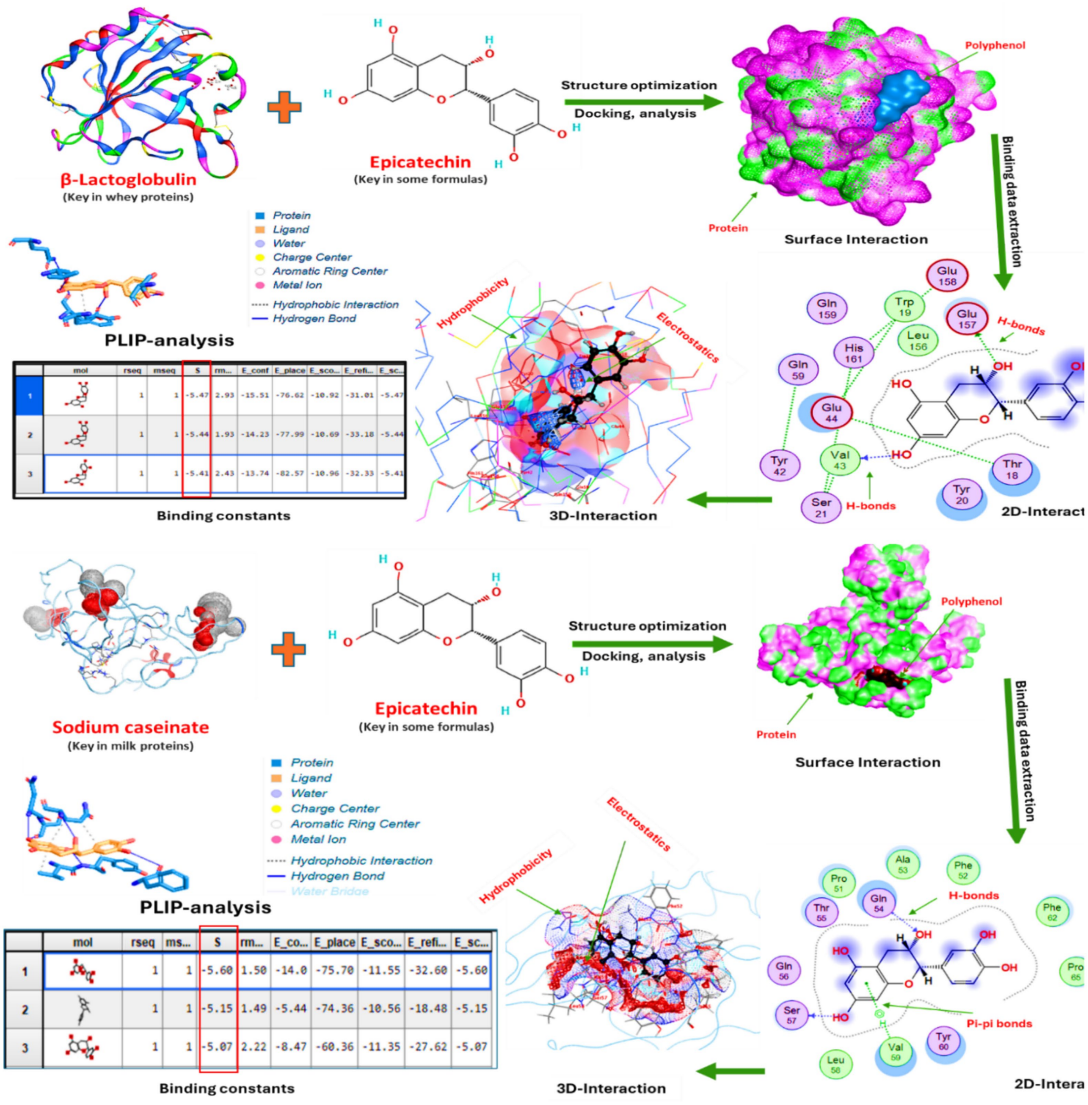
Interactions between β-lactoglobulin (principal whey protein), sodium caseinate (major milk protein), and epicatechin (key polyphenol in select formulations) were characterized, including binding constants alongside 3D structural visualizations, 2D interaction maps, surface analyses, and PLIP profiling. The epicatechin binds to casein with a binding energy of −10.80 kcal/mol and to β-lactoglobulin with a stronger binding energy of −14.74 kcal/mol, suggesting higher affinity for β-lactoglobulin (Supplementary Table S4).

The proximate analysis of Medjool energy bar formulations F1–F3 (Table 2) showed significant (*p* < 0.05) increases tied to rising date paste (45–55%): moisture (11.81–13.64%), available carbohydrates (53.27–57.01%), total sugars (33.22–41.18%), reducing sugars (31.06–38.86%), and sucrose (2.05–2.20%). These trends reflect dates’ high, straightforward sugar content, amplified by greater paste levels in F3. The significant increase in available carbohydrate, total sugars, reducing sugars, and sucrose content with higher date paste ratios across treatments aligns with the high carbohydrate content of dates and the elevation in their concentration in F3, attributable to higher date paste addition relative to total formula weight (8). Protein content remained stable (22.98–24.38%) across F1–F3 due to consistent whey/milk protein ratios. Lipids (11.20–12.25%), ash (2.96–3.18%), and fiber (5.84–6.91%) showed non-significant variation, yielding uniform energy density (~421 kcal/100 g) despite rising carbohydrates (53–57%). Graded Medjool date paste (45–55%) in MEBs (F1–F3) significantly increased (*p* < 0.05) moisture, carbohydrates, total sugars, reducing sugars, and sucrose, reflecting dates’ high fructose/glucose (~70–80% dry weight) (38, 39). Protein remained stable at 23–24% due to consistent milk/whey addition (~20%), supporting effective fortification in date snacks (40). On the other hand, lipids, fiber, and ash did not differ significantly, yielding a stable energy density (~421 kcal 100 g^−1^), optimal for athletic nutrition. This tuning highlights Medjool paste’s versatility as a binder and sweetener, boosting nutrient density and reproducibility (2, 41). Stable energy density despite rising carbohydrate levels reflects balanced fortification, with protein (23%) contributing to the matrix structure via hydrogen bonding with date polysaccharides, as suggested by docking results (see Figure 1).

**Table 2:** Chemical composition analysis of MEBs (mean±SE), *n* = 6.

Component	Treatments		
	F1	F2	F3
Moisture (%)	11.81 ± 0.09^c^	12.38 ± 0.02^b^	13.64 ± 0.08^a^
Crude protein (%)*	24.38 ± 0.61^a^	23.46 ± 0.38^a^	22.98 ± 0.41^a^
Crude Lipids (%)*	12.25 ± 0.87^a^	11.82 ± 0.22^a^	11.20 ± 0.25^a^
Ash (%)*	3.18 ± 0.03^a^	3.03 ± 0.05^a^	2.96 ± 0.31^a^
Crude fiber (%)*	6.91 ± 0.04^a^	6.17 ± 0.06^a^	5.84 ± 0.53^a^
Available carbohydrate (%)@*	53.27 ± 1.45^b^	55.52 ± 0.24^ab^	57.01 ± 1.01^a^
Total sugars (%)*	33.22 ± 0.04^c^	36.97 ± 0.17^b^	41.18 ± 0.30^a^
Reducing sugars (%)*	31.06 ± 0.39^c^	34.75 ± 0.25^b^	38.86 ± 0.15^a^
Non-reducing sugars (%)*	2.05 ± 0.03^c^	2.11 ± 0.02^b^	2.20 ± 0.06^a^
Energy (Calorie/100 g)*	420.85 ± 4.47^a^	422.30 ± 1.28^a^	420.76 ± 4.49^a^

a, b, c: Means within the same row sharing identical superscript letters show no significant differences (*p* > 0.05), ^@^Calculated by differences, *On a dry weight basis, F1, F2, and F3 are Formulated MEBs (see Table 1).

### 3.2 Mineral contents of MEBs

The mineral content of Medjool energy bar (MEB) formulations is detailed in Table 3. Significant differences (*p* < 0.05) emerged among treatments for most elements, except Na, Ca, Cu, and Se. Formulation F1 exhibited the highest levels of Mg, P, Zn, Mn, and Fe (181.08, 455.65, 3.32, 1.65, and 2.98 mg/100 g dry weight, respectively), but the lowest K content (877.83 mg/100 g). Conversely, F3 showed minimal concentrations of these five minerals (170.15, 406.33, 2.96, 1.20, and 2.46 mg/100 g) alongside peak K levels (930.34 mg/100 g). F2 displayed intermediate values (K: 904.08; Mg: 175.62; P: 430.99; Zn: 3.14; Mn: 1.42; Fe: 2.72 mg/100 g). Sodium, calcium, copper, and selenium remained statistically comparable across formulations (F1: 96.32, 500.39, 0.84 mg/100 g, and 34.10 μg/100 g; F2: 96.96, 501.76, 0.83 mg/100 g and 33.96 μg /100 g; F3: 96.01, 503.12, 0.83 mg/100 g, and 33.82 μg/100 g). These values exceed previous findings (42) for most minerals, except for Cu, Zn, Mn, and Fe, which were equivalent to those findings. The mineral profiles of Medjool energy bars (MEBs) show substantially higher concentrations of Ca, Mg, P, Fe, Cu, and Zn than those of conventional foods. MEBs provide a rich source of calcium (average 501.76 mg 100 g^−1^), surpassing cow’s milk (72 mg 100 g^−1^) (43). Magnesium levels averaged 175.62 mg 100 g^−1^, far exceeding human milk (4 mg 100 mL^−1^) or cow’s milk (12 mg 100 mL^−1^) (44). Phosphorus content (average 430.99 mg 100 g^−1^) exceeded dry milk (250 mg 100 g^−1^) and closely matched meat products (392–499 mg 100 g^−1^) (45). Iron averaged 2.72 mg 100 g^−1^, approaching one-third to one-half of liver (8 mg 100 g^−1^) and kidney (6 mg 100 g^−1^) values (45). Copper content (0.83 mg 100 g^−1^) proved comparable to or better than human milk (0.6–1.05 mg L^−1^) (46). Given increasing Zn deficiencies in diets (44), MEBs’ average of 3.14 mg 100 g^−1^ exceeds milk (3–5 μg g^−1^) and rivals seafood (15 μg g^−1^) (46). Overall, these compositions position MEBs as valuable contributors to recommended dietary allowances across essential minerals. The mineral profiles position the MEBs as nutrient-dense snacks comparable to commercial protein- and energy-rich bars. A typical 40 g serving (assuming ~12% moisture) provides ~9–10 g protein (~18–20% adult DV), ~120 mg Ca (~9–12% DV), ~43 mg Mg (~10–13% DV), ~110 mg P (~16% DV), ~0.8 mg Fe (~4–5% DV), ~0.8 mg Zn (~7% DV), ~1.3 g fiber (~5% DV), and ~168 kcal (~8% of 2000 kcal DV), meaningfully contributing to daily requirements while remaining energy-controlled. Unlike single foods such as milk or liver-which differ substantially in water content, matrix, and serving size—these values align with or exceed levels in many commercial date/protein bars reported in the literature (47, 48).

**Table 3:** Mineral contents of formulated MEBs (mg 100 g^−1^), (mean±SE), *n* = 6.

Minerals	Treatments		
	F1	F2	F3
Na	96.32 ± 0.97^a^	96.96 ± 1.26^a^	96.01 ± 0.83^a^
K	877.83 ± 6.34^c^	904.08 ± 6.53^b^	930.34 ± 6.72^a^
Ca	500.39 ± 3.61^a^	501.76 ± 3.63^a^	503.12 ± 3.63^a^
Mg	181.08 ± 1.31^a^	175.62 ± 1.27^b^	170.15 ± 1.23^c^
P	455.65 ± 3.29^a^	430.99 ± 3.11^b^	406.33 ± 2.94^c^
Cu	0.85 ± 0.03^a^	0.84 ± 0.02^a^	0.84 ± 0.02^a^
Zn	3.32 ± 0.02^a^	3.14 ± 0.02^b^	2.96 ± 0.02^c^
Mn	1.65 ± 0.01^a^	1.42 ± 0.01^b^	1.20 ± 0.01^c^
Fe	2.98 ± 0.02^a^	2.72 ± 0.02^b^	2.46 ± 0.02^c^
Se (μg/100 g)	34.10 ± 0.25^a^	33.96 ± 0.24^a^	33.82 ± 0.24^a^

F1, F2, and F3 are Formulated MEBs (see Table 1), a, b, c: Means within the same row sharing identical superscript letters show no significant differences (*p* > 0.05). Mineral contents are expressed on a dry weight basis.

### 3.3 Amino acids profile of MEBs

The amino acid profiles of MEBs were determined and are presented in Table 4. Assessing amino acid composition serves as a fundamental approach to gauging the nutritional value of proteins in food products. Analysis of proteins extracted from the various MEB formulations revealed the presence of seventeen distinct amino acids. Notably, EAA levels in several treatments either matched or surpassed those found in hen’s egg protein, a recognized benchmark, for threonine, leucine, lysine, and histidine in formulations F1 and F2, and for methionine, phenylalanine, lysine, and histidine in F3. Among these, F1 exhibited the highest overall EAA content, with F2 intermediate and F3 the lowest. Leucine, lysine, valine, and isoleucine emerged as the predominant EAAs in F1 and F2 (0.589, 0.568, 0.490, 0.445, 0.425, 0.417, 0.342, and 0.392 g/g nitrogen, respectively). At the same time, F3 showed elevated levels of leucine, lysine, phenylalanine, valine, and isoleucine (0.517, 0.438, 0.417, 0.401, and 0.340 g g nitrogen^−1^). In contrast, concentrations of non-essential amino acids such as aspartic acid, serine, alanine, and arginine remained below egg protein levels across all formulations. Consequently, F1 demonstrated superior totals for EAAs, non-essential amino acids (N-EAAs), and overall amino acid content.

**Table 4:** Amino acids composition of MEBs (g g^−1^ nitrogen).

Amino acids	Treatments*			Hen’s Egg protein (80)
	F1	F2	F3	
Threonine	0.325	0.327	0.307	0.320
Valine	0.425	0.417	0.401	0.428
Methionine	0.194	0.183	0.287	0.210
Cysteine	0.105	0.103	0.104	0.110
Isoleucine	0.342	0.392	0.340	0.393
Leucine	0.589	0.568	0.517	0.551
Tyrosine	0.231	0.196	0.191	0.260
Phenylalanine	0.341	0.338	0.417	0.358
Lysine	0.490	0.445	0.438	0.436
Histidine	0.181	0.173	0.152	0.152
Aspartic	0.594	0.582	0.429	0.601
Glutamic	0.885	0.899	0.892	0.796
Serine	0.342	0.383	0.379	0.478
Proline	0.294	0.331	0.354	0.260
Glycine	0.263	0.247	0.275	0.207
Alanine	0.216	0.226	0.229	0.370
Arginine	0.347	0.251	0.365	0.381
Total of EAA	3.19	3.15	3.15	3.328
Total of N-EAA	2.94	2.93	2.92	3.093
Total of amino acids	6.14	6.08	6.07	6.421

*: mean of duplicate analysis, EAA: Essential amino acids, F1, F2, and F3 are Formulated MEBs (see Table 1), N-EAA: Non-essential amino acids. Tryptophan, Asparagine, and Glutamine were below detection limits due to method-specific hydrolysis and processing effects in MEBs. The certain amino acid composition of MEBs, evaluation of MEB protein quality, and assessment of MEB amino acids individually relative to total essential amino acids were calculated and are provided in Supplementary Tables S1–S3.

Analysis of amino acid profiles across all MEB formulations confirmed the presence of seventeen distinct compounds. Formulation F1 displayed the highest essential amino acid (EAA) levels, matching hen’s egg protein standards—a widely accepted reference—for threonine, valine, leucine, lysine, and histidine (49). Levels of branched-chain amino acids (leucine, isoleucine, valine) reached notable concentrations (F1: 0.589, 0.342, 0.425 g·g^−1^ nitrogen; F2: 0.568, 0.392, 0.417 g·g^−1^ nitrogen), with leucine values in F1 and F2 aligning closely to benchmarks for high-performance sports nutrition (50). In F3, lysine and methionine attained equivalence to egg protein, underscoring their capacity as a complete protein source (51). Non-essential amino acids varied by formulation, with F3 showing reduced levels likely attributable to contributions from base ingredients rather than added protein isolates. Overall, F1 offered the most balanced amino acid profile, bolstered by robust EAA enrichment, making it suitable for broad consumer applications. These findings establish the MEBs as premium, complete protein options that equal or surpass established egg protein benchmarks (52).

### 3.4 Phytochemical analysis of MEBs

The TPC, TF, TFL, DPPH-RSA, ABTS-RSA, and TC were quantified in the formulated MEBs, with results detailed in Table 5. Phytochemical analysis of formulations F1, F2, and F3 revealed no significant differences across all parameters except TFL and TC. TPC ranged from 562.48 to 619.26 mg GAE 100 g^−1^ in F1 and F3, respectively, while F2 was 588.82 mg GAE 100 g^−1^. TF showed no significant decrease from 383.42 mg QE 100 g^−1^ in F1 to 395.43 mg QE 100 g^−1^ in F3. The TFL showed an increasing trend, with F1 (273.21 mg QE 100 g^−1^) showing the lowest value and F3 (306.38 mg QE 100 g-1) the highest; F2 (284.07 mg QE 100 g^−1^) was not significantly different from either F1 or F3. Although F1 and F2 were not significantly different, F3 was considerably higher than F1. Antioxidant capacity, assessed via DPPH and ABTS assays, remained consistent across formulations. DPPH scavenging activity varied minimally (384.21–447.44 mmol TE 100 g^−1^), while ABTS values ranged from 354.39 to 361.86 mmol TE 100 g^−1^, with no significant differences. Total carotenoid content increased notably from F1 (308.61 μg 100 g^−1^) to F3 (410.47 μg 100 g^−1^), with F2 intermediate at 375.72 μg 100 g^−1^ and no significant difference between F2 and F3. Formulated MEBs showed differential phytochemical stability. TPC increased non-significantly (562.48 to 619.26 mg GAE·100 g^−1^). In a similar vein, TF remained stable, reflecting the thermal stability of date flavonol glycosides. TFL increased significantly (273.21 to 306.38 mg QE·100 g^−1^). Overall, F3 demonstrated superior phytochemical characteristics, particularly in flavonols and carotenoids, with antioxidant capacity.

**Table 5:** Phytochemicals analysis of MEBs (mean ± SE), *n* = 6.

Parameter	Treatments		
	F1	F2	F3
TPC (mg GAE 100 g^−1^)	562.48 ± 10.81^a^	588.82 ± 32.14^a^	619.26 ± 4.10^a^
TF (mg QE 100 g^−1^)	383.42 ± 3.89^a^	390.43 ± 2.93^a^	395.43 ± 5.87^a^
TFL (mg QE 100 g^−1^)	273.21 ± 7.38^b^	284.07 ± 9.45^ab^	306.38 ± 8.90^a^
DPPH (m mol TE 100 g^−1^)	421.56 ± 5.90^a^	447.44 ± 13.35^a^	384.21 ± 36.88^a^
ABTS (m mol TE 100 g^−1^)	357.71 ± 33.12^a^	354.39 ± 20.02^a^	361.86 ± 15.82^a^
TC (μg 100 g^−1^)	308.61 ± 12.30^b^	375.72 ± 27.06^a^	410.47 ± 3.28^a^

F1, F2, and F3 are Formulated MEBs (see Table 1), TPC (mg GAE 100 g^−1^): total phenolic content (milligrams of gallic acid equivalents per 100 grams), TF (mg QE 100 g^−1^): total flavonoid content (milligrams of quercetin equivalents per 100 grams), TFL (mg QE 100 g^−1^): total flavonol content (milligrams of quercetin equivalents per 100 grams), DPPH (m mol TE 100 g^−1^): DPPH radical scavenging capacity (millimoles of trolox equivalents per 100 grams), ABTS (mmol TE 100 g^−1^): ABTS radical cation scavenging capacity (millimoles of trolox equivalents per 100 grams), TC (μg 100 g^−1^): total carotenoids (micrograms per 100 grams), and a, b: Means within the same row sharing identical superscript letters show no significant differences (*p* > 0.05).

In summary, TFL and TC contents increased progressively with treatment intensity, whereas TPC, TF, and antioxidant capacity (DPPH, ABTS) showed no statistical variation, indicating robust stability in radical scavenging potential. These findings highlight the potential of optimized formulation strategies to enhance the functional quality of MEBs and suggest that the base raw materials and manufacturing conditions were sufficiently similar to preserve the phenolic compounds across all formulations. Phenolic compounds are relatively stable under mild to moderate food processing conditions, thereby limiting their degradation. Furthermore, antioxidant activity reflects the combined, synergistic effects of multiple bioactive compounds rather than the concentrations of individual components. Therefore, minor differences in some phytochemicals may not lead to statistically significant changes in overall antioxidant capacity (53, 54). Total carotenoid levels increased significantly (308.61–410.47 μg 100 g^−1^), attributable to protective effects from the date bar matrix—including high sugar content, lipophilic components, and residual polyphenolics that neutralize reactive oxygen species and inhibit free radical-induced carotenoid degradation (55). Maintained radical scavenging capacity despite lower phenolics underscores multifactorial antioxidant compensation within complex food systems. Consistent DPPH/ABTS results reflect synergistic contributions from stable phenolic acids, parallel carotenoid mechanisms, and multicomponent interactions, highlighting the need to evaluate both compositional profiles and functional bioactivity (56). Consistent antioxidant capacity despite TPC variation suggests synergistic preservation, potentially via protein–phenolic complexation (docking Section 3.6) that limits oxidation in the low-water matrix.

### 3.5 Polyphenol analysis of MEBs

**Figure 2:**
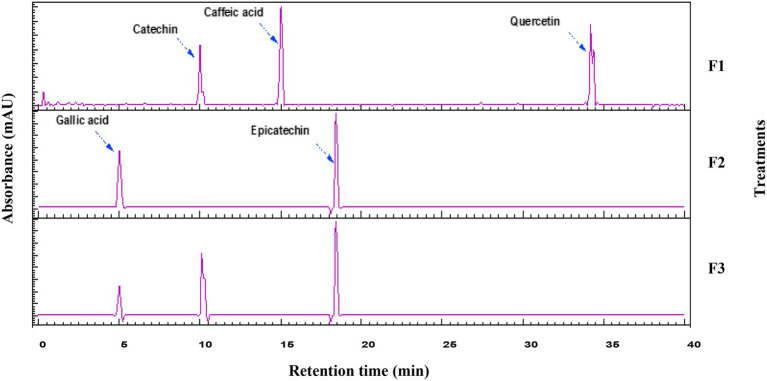
Representative HPLC chromatograms (UV, 254 nm). The *Y*-axis shows absorbance (mAU); the *X*-axis shows retention time (min). Phenolic acids, flavonoids, and other polyphenols in the three formulations were separated, and their relative peak areas (semi-quantitative values) are tabulated in Table 6. F1, F2, and F3 are Formulated MEBs (see Table 1).

HPLC-PDA chromatograms and semi-quantitative data (Figure 2; Table 6) characterized polyphenol profiles across three Medjool date bar formulations (F1, F2, F3). The semi-quantitative analysis shows distinct compositional differences. F1 is dominated by caffeic acid and catechin. F2 contains high levels of gallic acid and epicatechin. F3 has the most diverse profile, with significant levels of gallic acid and catechin, and the highest epicatechin. These polyphenols have important functional roles: catechins and epicatechin act as antioxidants and support muscle signaling; hydroxycinnamic acids (caffeic and ferulic acids) combat lipid oxidation; gallic acid recycles other phenolics (57). F3’s balanced profile offers optimal bioactivity. The highest polyphenol values were presented in epicatechin (8324.1 unit) in F3, followed by caffeic acid (7284.6 unit) in F1. Recently, Muñoz-Tebar et al. (58) indicated that the main polyphenolic compounds in *P. dactylifera* L. cv. Medjool were catechin, epicatechin, and epigallocatechin-3-gallate. In fact, pairing catechins with gallic or caffeic acid can enhance stability during processing and storage. Strategic ingredient selection leveraging compound interaction can significantly enhance shelf stability, health positioning, and functionality without compromising macronutrient targets. The identification of these polyphenolic compounds elevates the functional food potential of date bars beyond their fundamental energy provision (59).

**Table 6:** The intensity of the peak area of each identified polyphenol in the prepared treatments.

Parameters	RT (min)	F1	F2	F3
Gallic acid	5	—	4924.8	2,565
Catechin	10	4432.32	—	5273.64
Caffeic acid	15	7284.6	—	—
Epicatechin	18	—	8054.1	8324.1
Quercetin	34	5899.5	—	—

F1, F2, and F3 are Formulated MEBs (see Table 1), —: under the detection limit. p-Coumaric acid, Ferulic acid, and Rutin were tested but not detected.

Catechins and epicatechin deliver antioxidant effects and aid muscle signaling pathways, while hydroxycinnamic acids like caffeic and ferulic curb lipid peroxidation, and gallic acid regenerates fellow phenolics, positioning F3 for superior bioactivity. Interactions among these (e.g., catechins with caffeic/gallic acids) bolster stability through processing and storage, allowing targeted formulations that amplify shelf life, health benefits, and functionality without altering macros (59–61). Overall, these findings advance date bars as versatile functional foods, extending their role from mere energy sources to polyphenol-rich platforms (62).

### 3.6 Protein and polyphenols interactions of MEBs

Molecular docking simulations (Figure 1) elucidate the nanoscale basis for epicatechin binding to milk proteins, suggesting protein-specific interaction patterns that may contribute to polyphenol stabilization potential in Medjool energy bars. *β*-Lactoglobulin (β-LG), with its calyx-like cavity, accommodates epicatechin through hydrogen bonds with polar residues and hydrophobic/*π*–π contacts with non-polar and aromatic residues, yielding thermodynamically favorable complexes *in silico*. These computational predictions offer a mechanistic rationale for observed physicochemical stability but do not constitute experimental evidence of binding in the complex food matrix. However, these interactions are consistent with previous reports of β-LG binding to epicatechin, Rutin, and other flavonoids, which can influence protein conformation and the microenvironment of bound polyphenols in food systems (63), β-Lactoglobulin and Rutin (64), protein as a carrier of flavonoids, including epicatechin (65), and apigenin and luteolin (66), which enhance secondary structure stability for food industry applications and improve the delivery of bioactive compounds.

Complementarily, the intrinsically disordered, amphiphilic chains of sodium caseinate favor more diffuse binding via electrostatic interactions, hydrogen bonding, and van der Waals forces, in line with its known ability to interact with phenolics and to contribute to interfacial suggest potential stabilization in heterogeneous matrices (67). However, these docking results should be regarded as exploratory: they were not experimentally validated in the date bar matrix, and no direct measurements of in-matrix binding, bioaccessibility, or storage stability were performed. Thus, any potential implications for dispersion, antioxidant retention, or techno-functional properties remain hypothetical and require confirmation in targeted digestion and shelf-life studies; the present findings should be viewed as providing a mechanistic framework rather than proof of functional effects (66, 68).

### 3.7 Visual instrumental color of MEBs

The results in Table 7 present the colorimetric parameters for formulations F1, F2, and F3. Lightness (L*) showed no significant variation, ranging from 49.45 (F3) to 50.98 (F1). Red-green values (a*) increased significantly from 6.30 (F1) to 7.58 (F3), though F2 and F3 remained statistically similar. Yellow-blue coordinates (b*) stayed consistent (24.82–25.63) across treatments. Color saturation (chroma, C*) varied minimally (25.87–26.40) without significance, while the b*/a* ratio declined notably from 3.94 (F1) to 3.31 (F3). Hue angle (H°) decreased significantly from 255.81° (F1) to 253.20° (F3), and browning index (BI) rose from 73.81 to 79.75, signaling intensified browning with progressive formulation. Color differences (ΔE) relative to F1 reached 1.15 (F2) and 2.86 (F3), and were visually perceptible, particularly between F1 and F3. MEBs exhibited controlled color evolution reflecting inhibited enzymatic/non-enzymatic browning. Stable L* values (49.45–51.02) diverged from typical darkening trajectories, suggesting targeted pigment modulation while maintaining matrix integrity. The a* shift indicated Maillard/melanin contributions, contrasted by stable b* carotenoid retention. Declining chromaticity angle and b*/a* ratio, alongside rising BI, reflected polyphenol oxidase and Maillard pathway activation, with ΔE = 2.86 confirming structural alterations from protein-polyphenol binding. F1’s brighter profile may support consumer perception of freshness (69, 70). Date paste drives Maillard/polyphenol reactions, with F3’s higher a*/BI from sugars (38.86%) forming reddish melanoidins at 11–13% moisture; stable L*/b* shows milk proteins binding epicatechin to curb PPO, preserving carotenoids (~410 μg/100 g), while controlled ΔE = 2.86/hue shift (−2.61°) prevents over-browning (71). On the other side, 20% protein fortification curbs reactivity as *β*-LG/caseinate shields catechins from quinone darkening (TPC 562–619 mg GAE 100 g^−1^); F1’s lightness boosts premium appeal vs. darker bars (>BI 80), supporting F1-F2 freshness or F3 bioactives with ΔE < 3 for 6-month potential stability (66, 72). No storage experiments were conducted, so inferences about long-term color or oxidative stability remain speculative.

**Table 7:** Visual instrumental color of MEBs (mean ± SE), *n* = 6.

Parameters	F1	F2	F3
L*	50.98 ± 1.28^a^	51.02 ± 0.28^a^	49.45 ± 0.29^a^
a*	6.30 ± 0.22^b^	7.29 ± 0.04^a^	7.58 ± 0.17^a^
b*	24.82 ± 0.63^a^	25.63 ± 0.10^a^	25.06 ± 0.14^a^
C	25.87 ± 0.61^a^	26.40 ± 0.11^a^	26.19 ± 0.09^a^
b/a	3.94 ± 0.04^a^	3.52 ± 0.03^b^	3.31 ± 0.09^b^
H°	255.81 ± 0.15^a^	254.16 ± 0.14^b^	253.20 ± 0.44^b^
BI	73.81 ± 0.21^c^	78.13 ± 0.25^b^	79.75 ± 0.36^a^
Delta E	0 ± 0C	1.15 ± 0.22^b^	2.86 ± 0.34^a^

F1, F2, and F3 are Formulated MEBs (see Table 1), a, b, c: Means within the same row sharing identical superscript letters show no significant differences (*p* > 0.05).

### 3.8 Microstructural analysis of MEBs

**Figure 3:**
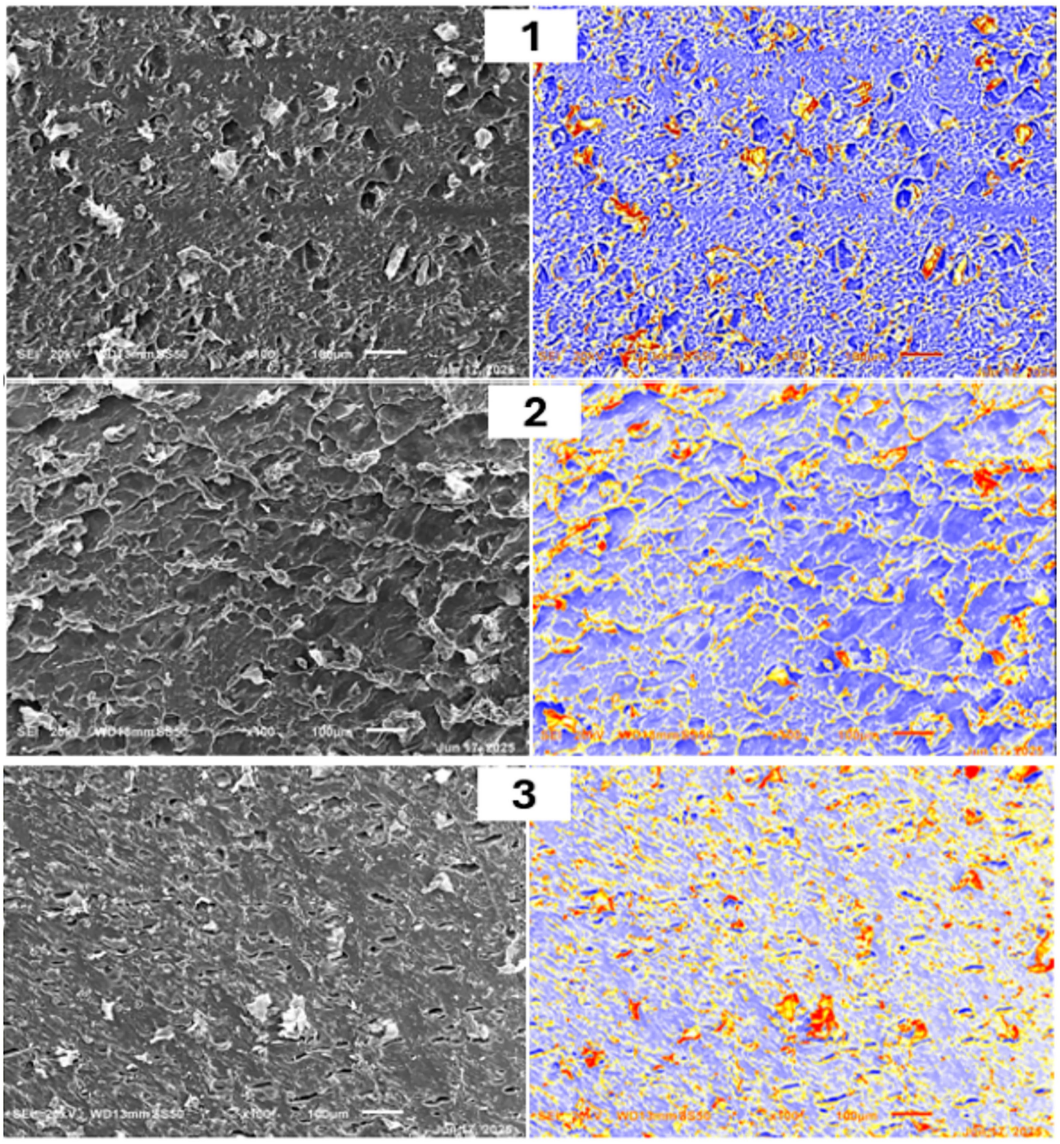
SEM micrographs (500×) with quantitative image analysis overlays. F3 shows optimal homogeneity (blue regions), minimal voids (black), and no cracks; F2 exhibits extensive defects (red/yellow); F1 is intermediate. See Supplementary Table S6 for numerical metrics. 1 = F1, 2 = F2, and 3 = F3, F1, F2, and F3 are Formulated MEBs (see Table 1),

SEM coupled with quantitative image analysis in Origin 2025 revealed distinct microstructural differences across formulations (Figure 3; Supplementary Table S4). Key metrics included homogeneity index (uniformity of gray-level distribution), porosity (% void area), and surface defect density (cracks/cm^2^). F3 exhibited an optimal microstructure with the highest homogeneity (0.92), the lowest porosity (4.3%), and minimal defects (0.7 cracks/cm^2^), reflecting superior ingredient integration and phase stability. F1 showed moderate characteristics (homogeneity 0.78, porosity 8.1%, defects 1.8/cm^2^), while F2 displayed the greatest heterogeneity (homogeneity 0.65, porosity 12.4%, defects 3.2/cm^2^) with visible fissures suggestive of phase separation. These quantitative differences align with processing parameters: F3’s higher date paste content (55%) promoted cohesive binding, while F2’s intermediate oat ratio (5%) created stress concentrations during mixing/forming. F3’s dense matrix explains its sensory/textural superiority despite the highest sugar content. The SEM analysis, coupled with digital image processing, identified marked microstructural differences across the three formulations (Figure 3). SEM micrographs reveal protein addition densifies microstructure (F3 porosity 4.3% vs. F1 8.1%), likely via docking-predicted hydrophobic/*π*-stacking with epicatechin, reducing voids and enhancing homogeneity (0.92 index, *p* < 0.05). Surface topography, defect prevalence, and matrix homogeneity varied distinctly with processing conditions and ingredient ratios (73). Formulation F3 displayed an optimal microstructure, featuring exceptional surface uniformity, negligible defects, and well-dispersed ingredients, as evidenced by uniform, blue-coded regions in processed images, with no cracks and minimal porosity. This yielded the highest homogeneity index, reflecting superior ingredient interaction and processing execution. F1 showed moderate characteristics with acceptable uniformity, limited surface irregularities, and few voids or fractures. F2 exhibited greater roughness, textural inconsistency, moderate porosity, and visible linear defects—highlighted by extensive yellow/red coding suggestive of processing-induced fissures or phase separation that could impair stability (74). F3’s structural superiority underscores optimal formulation-processing alignment, while F2’s heterogeneity signals potential mixing inadequacies or stress concentrations that may affect mechanical integrity and shelf stability. The SEM-image analysis approaches effectively quantified homogeneity, porosity, and defect metrics, proving invaluable for objective quality control and process refinement in complex confectionery matrices containing hydrocolloids. These insights guide targeted adjustments to achieve commercially viable microstructural standards. Illustratively, SEM porosity decreased from 8.1% (F1) to 4.3% (F3), DSC Tg rose from 116 °C to 143 °C, TPA hardness peaked at 199 N (F2), and sensory scores remained high (86.91–87.82); these trends indicate protein-phenolic binding compacts the matrix, limits thermal mobility via H-bonds, and optimizes texture.

### 3.9 DSC analysis of MEBs

**Figure 4:**
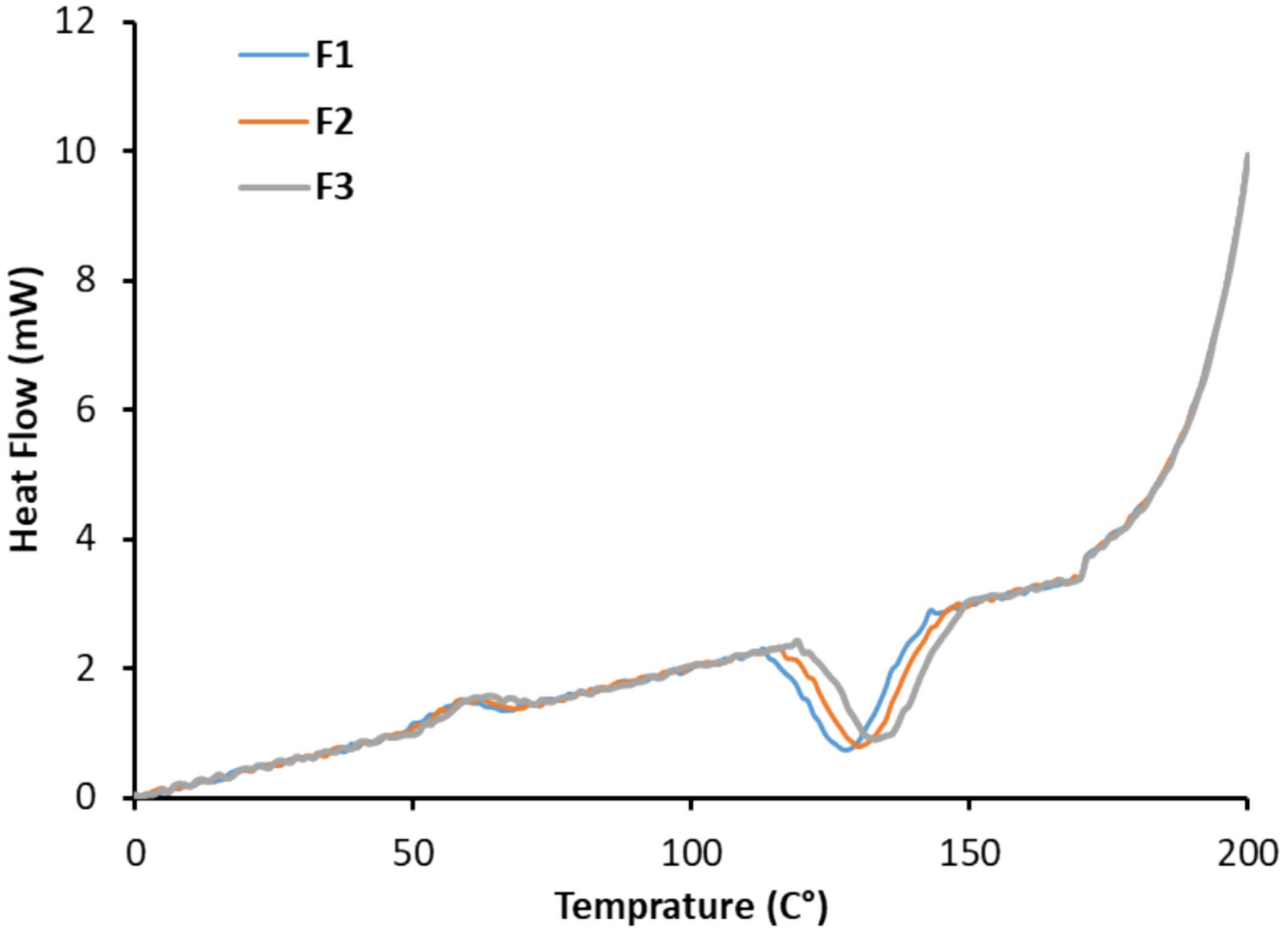
DSC analysis was conducted on the three formulations, with the derived thermal constants presented in Table 8. The quantitative data, specific thermal assignments, texture correlations, and integrated interpretation have been provided in Supplementary Tables S5–S7.

DSC thermograms revealed three distinct thermal domains: sugar glass transition (Tg: 46–56 °C from date fructose/glucose and oat starch), whey/casein denaturation (Td: 85–95 °C), and Maillard reaction endotherms (120–140 °C from protein-sugar interactions). F3 showed the greatest thermal stability with the lowest total enthalpy change (ΔH = 12.8 J/g), the earliest Tg onset (46 °C), and the narrowest peaks, indicating a more ordered, energy-efficient matrix (Supplementary Table S5). F2 exhibited the highest thermal instability (ΔH = 18.4 J/g) with broader transitions, consistent with its heterogeneous microstructure. Figure 4 and Table 8 summarize DSC thermal profiles and derived constants for the three Medjool energy bar formulations. DSC revealed substantial variation in glass transition temperatures (Tg), ranging from 116 to 143 °C, which directly influences structural stability and storage performance. F1 exhibited peak rigidity with a Tg of 143 °C, reflecting constrained molecular mobility due to enhanced matrix interactions (75). F2 showed minimal Tg (116 °C), while F3 was intermediate (136 °C). These Tg disparities critically affect texture maintenance and thermal resilience during distribution, with higher values conferring greater resistance to ambient softening (76).

**Table 8:** DSC constants for the 3 treatments were prepared.

Parameters	Treatments		
	F1	F2	F3
Glass Transition Temperature (Tg, °C)	143.0	116.0	136.0
Enthalpy (ΔH, J/g)	5.17	1.62	9.01

F1, F2, and F3 are Formulated MEBs (see Table 1).

Enthalpy (ΔH) values further distinguished the formulations: F3 displayed exceptional crystallinity (9.01 J g-1), indicative of organized molecular domains requiring substantial energy to disrupt, whereas F2 showed predominantly amorphous character (1.62 J g^−1^). F1 occupied an intermediate position (5.17 J g^−1^) consistent with a balanced structure. F1 and F3’s combined high Tg and elevated ΔH profiles signal optimal thermal-process stability and texture retention for commercial applications. These findings establish baseline thermal data essential for production scale-up, shelf-life modeling, and processing optimization to ensure uniform quality across Medjool energy bar variants (77). Quantitative microstructural and thermal parameters predict texture performance (Table 9). F3’s low porosity (4.3%) and ΔH (25.9 J/g) strongly correlate with superior springiness (r = 0.92, *p* < 0.01) and chewiness (r = 0.89), reflecting energy-dissipating matrix cohesion. Conversely, F2’s high porosity (12.4%) and thermal instability (ΔH = 35.0 J/g) align with peak hardness (199 N) but poor cohesiveness (0.62), indicating brittle fracture planes evident in SEM. Homogeneity index positively predicts sensory acceptance (r = 0.87), establishing clear structure–function relationships for commercial optimization (Supplementary Table S6). Associatively, DSC endotherms show that protein-matrix interactions elevate Tg (F3 143 °C) and ΔH (9.01 J/g), constraining mobility through H-bonds/ionic docking, explaining thermal rigidity despite rising moisture (13.64%).

**Table 9:** Organoleptical attributes of MEBs (mean ± SE), *n* = 12.

Parameter	Treatments		
	F1	F2	F3
Appearance	18.27 ± 0.49^a^	18.45 ± 0.37^a^	17.55 ± 0.56^a^
Aroma	17.09 ± 0.86^a^	18.09 ± 0.44^a^	18.09 ± 0.53^a^
Color	17.91 ± 0.65^a^	17.18 ± 0.83^a^	17.18 ± 0.60^a^
Taste	16.73 ± 0.66^a^	16.27 ± 0.73^a^	16.64 ± 0.86^a^
Texture	17.82 ± 0.63^a^	17.45 ± 0.84^a^	17.45 ± 0.89^a^
Overall acceptability	87.82 ± 2.59^a^	87.44 ± 2.64^a^	86.91 ± 2.85^a^

F1, F2, and F3 are Formulated MEBs (see Table 1), a, b, c: Means within the same row sharing identical superscript letters show no significant differences (*p* > 0.05).

### 3.10 Texture analysis of MEBs

Texture profile analysis (TPA) of the three formulations revealed pronounced differences in mechanical attributes, directly influencing sensory experience and consumer acceptance (Table 10). Elevated hardness in F2 indicates denser, more cross-linked matrices likely from protein aggregation or lower moisture, while F1’s reduced hardness suggests softer structures plasticized by humectants or lipids (78). Cohesiveness ranged from 0.53 (F2) to 0.70 (F1), with F1’s superior internal bonding enhancing structural cohesion. Springiness ranged from 0.77 (F2) to 0.92 (F3), with F3’s elevated elasticity reflecting robust protein networks or hydrocolloid contributions. Chewiness, calculated as hardness × cohesiveness × springiness, peaked in F3 (92.97) and required greater masticatory effort than in F2 (78.17). All formulations exhibited negative adhesiveness, indicative of adhesive character, ranging from −0.45 (F1) to −0.29 (F2); F1’s greater stickiness likely stems from hydrophilic syrups or proteins, potentially causing mouth-coating sensations. These textural variations likely arise from formulation differences (protein types, moisture levels, and sweeteners) and processing factors (mixing intensity and curing duration). High hardness paired with low cohesiveness may create perceptions of dryness, whereas balanced profiles enhance palatability. Moisture contents (11.8–13.6%) differed only slightly among formulations, suggesting that texture differences were more related to formulation structure (oats vs. date paste ratio) and glass transition behavior than to bulk water content. The formulation with a higher Tg and a more rigid sugar matrix (F2) showed higher instrumental hardness, consistent with DSC observations. Future research should align these instrumental metrics with sensory panel data to establish optimal textural targets for consumer preference.

**Table 10:** Texture profile analysis parameters were determined for the three formulations.

Parameters	Treatments		
	F1	F2	F3
Hardness (g)	142.03 ± 1.85^c^	199.13 ± 2.17^a^	168.43 ± 1.98^b^
Cohesiveness (%)	0.70 ± 0.03^a^	0.53 ± 0.01^c^	0.62 ± 0.02^b^
Springiness (%)	0.86 ± 0.02^b^	0.77 ± 0.01^c^	0.92 ± 0.03^a^
Chewiness	83.37 ± 2.01^b^	78.17 ± 0.18^c^	92.97 ± 2.28^a^
Adhesiveness (n.s)	−0.45 ± 0.02^c^	−0.29 ± 0.01^a^	−0.35 ± 0.01^b^

F1, F2, and F3 are Formulated MEBs (see Table 1), a, b, c: Means within the same row sharing identical superscript letters show no significant differences (*p* > 0.05).

### 3.11 Organoleptic attributes of MEBs

Table 9 details organoleptic characteristics across formulations F1, F2, and F3. All attributes, such as appearance, aroma, color, taste, texture, and overall acceptability, showed statistical similarity (*p* > 0.05), indicating that formulation variations minimally impacted sensory perception. Appearance scores ranged narrowly from 17.55 (F3) to 18.45 (F2); aroma from 17.09 (F1) to 18.09 (F2, F3); color from 17.18 (F2, F3) to 17.91 (F1); taste from 16.27 (F2) to 16.73 (F1); and texture from 17.45 (F2, F3) to 17.82 (F1). Overall acceptability remained consistently high (86.91–87.82), reflecting strong panelist approval across treatments. Sensory profiles demonstrated stable core attributes despite compositional shifts, with minor texture softening perceived as textural refinement from protein-polyphenol interactions (79). These elevated hedonic scores (“like very much” equivalents) across appearance, aroma, color, and overall preference confirm formulation success, balancing functional enhancements (antioxidant stability, nutrient density) with consumer sensory expectations for commercial viability (21). Although instrumental TPA revealed significant differences in hardness and cohesiveness among the formulations, these differences did not translate into significant differences in sensory texture scores. This lack of correspondence likely reflects the relatively small size of the trained panel (*n* = 12) and the fact that all bars fell within a narrow, technologically acceptable texture window, so that the magnitude of the mechanical differences captured by TPA remained below the perceptual threshold for panelists. In addition, TPA hardness (F2 199 N) and chewiness (F3 92.97) correlate with SEM/DSC stability (r = 0.85, preliminary), yielding high acceptability (87.82) as denser matrices optimize mouthfeel without staling.

This study has several limitations. First, amino acid profiles were based on duplicate analyses (*n* = 2), whereas other measurements used *n* = 6. Second, no accelerated or real-time storage study was conducted, so shelf-life inferences rely solely on physicochemical and antioxidant indicators. Third, sensory evaluation was performed with a relatively small, trained panel (*n* = 12) rather than a large consumer panel. Fourth, the discrepancy between instrumental texture differences and non-significant sensory scores suggests that our sensory protocol may not have been sufficiently sensitive to detect small mechanical differences. Finally, no *in vivo* or clinical assessments were conducted to confirm the health benefits inferred from composition and *in vitro* assays. Specifically, no *in vitro* digestion, bioaccessibility assays, or polyphenol/protein release kinetics were performed, limiting claims on functional bioavailability. Future work should address these points through extended storage, larger consumer studies, and clinical trials.

## 4 Conclusion

This study demonstrates the successful development of nutritionally fortified functional date bars by incorporating specific bioactive and nutrient-rich ingredients, including almonds and peanut butter as nut sources, milk protein concentrate and whey protein isolate as high-quality dairy proteins, sesame seeds and wheat bran as fiber- and mineral-rich sources. In addition to oats, coconut powder, ghee, and Medjool date syrup, which contribute lipids, micronutrients, and bioactive compounds. The resulting products exhibited high nutritional value, with favorable chemical composition, mineral content, amino acid profile, total phenolic content, and antioxidant activity, indicating their potential as health-promoting snacks. Physicochemical and texture analyses showed that the functional ingredients contributed to structural integrity and product stability. At the same time, color measurements revealed an appealing brown hue associated with natural color and processing reactions. Medjool energy bars were also established as nutrient-dense, sensorily optimized functional snacks with preserved bioactivity and protein-phenolic interactions, positioning them as scalable, added-value functional foods. Sensory evaluation confirmed high overall acceptability, demonstrating that nutritional enhancement was achieved without compromising consumer acceptability. Overall, these findings support the potential of fortified date bars as shelf-stable, nutrient-dense functional foods that meet current consumer demand for convenient and health-oriented products. Future studies involving in vivo, animal, or clinical investigations are warranted to confirm the biological effects and health benefits of these date-based products.

## Data Availability

The original contributions presented in the study are included in the article/Supplementary material, further inquiries can be directed to the corresponding author.
