# Supplementary material for: Development, nutritional profiling, physicochemical properties, computational protein-polyphenol interactions, and sensory properties of high-energy Medjool date bars
Source: Front Nutr. 2026 May 11;13:1813284. doi: 10.3389/fnut.2026.1813284 (PMC13214981; doi:10.3389/fnut.2026.1813284)
Supplement: Supplementary file 1 [file Data_Sheet_1.PDF]

Table S1. Scores of essential amino acids to limiting the three essential amino acids responsible for limiting the quality of protein of Medjool high-carb formula bars (MCBs).

Amino Acid	F3	F2	F3	Suggested Amino Acid Pattern
**Threonine**	132.33	134.46	126.44	40
**Valine**	138.44	137.17	132.13	50
**Isoleucine**	139.25	161.18	140.03	40
**Leucine**	137.04	133.46	121.68	70
**Tyrosine**	107.39	92.11	89.90	35
**Phenylalanine**	115.70	115.82	143.12	48
**Histidine**	140.38	135.50	119.24	21
**Lysine**	145.10	133.07	131.20	55
**Methionine + Cyst.**	108.20	104.53	106.58	45
**First Limiting AA**	Tyrosine	Tyrosine	Tyrosine	
**Second Limiting AA**	Methionine + Cyst.	Methionine + Cyst.	Methionine + Cyst.	
**Third Limiting AA**	Phenylalanine	Phenylalanine	Histidine	

*According to FAO/WHO AD HOC Committee (FAO, 1973).

$$\text{Amino acid score according to FAO } (1973) = \frac{\text{mg amino acid in 1 g protein}}{\text{mg amino acid sugested by FAO/WHO}} \times 100$$

Table S2. The Nutritional Evaluation of Medjool high-carb formula bars (MCBs) Proteins

Treatments	T.EAA (g/16N)	T.N-EAA (g/16N)	E:N Ratio	E:P Ratio	E:T Ratio	EAAI (%)
**F1**	51.052	47.040	1.085	0.511	0.521	102.10
**F2**	50.240	46.720	1.075	0.502	0.518	99.70
**F3**	50.400	46.720	1.079	0.504	0.519	97.80
**Hen's Egg FAO (1970)**	53.248	49.488	1.076	0.533	0.518	100.00
**Beef FAO (1970)**	42.727	57.276	0.746	0.427	0.427	79.55

E:N ratio of essential amino acids to non-essential amino acids; E:T ratio of essential amino acids to total amino acids; E:P ratio of essential amino acids to protein; EAAI-Essential amino acid index according to **Oser (1959)**.

Table S3. Assessment of individual amino acids (A) to reference essential amino acids (E) in hen's egg protein (mg individual AA/g TEAA) of Medjool high-carb formula bars (MCBs).

Amino Acids	F1	F2	F3	Hen's Egg FAO (1970)
**Threonine**	101.88	103.81	97.46	110.42
**Valine**	133.23	132.38	127.30	147.69
**Isoleucine**	107.21	124.44	107.94	135.61
**Leucine**	184.64	180.32	164.13	190.13
**Tyrosine**	72.41	62.22	60.64	89.72
**Phenylalanine**	106.90	107.30	132.38	123.53
**Histidine**	56.74	54.92	48.24	52.45
**Lysine**	153.61	141.27	139.05	150.45
**Methionine + Cyst.**	93.73	90.79	124.13	151.00

A/E Ratio = mg amino acid per grams total essential amino acids.

Table S4. Molecular Docking Analysis of Epicatechin Binding to Casein and β-Lactoglobulin: Binding Energies and Key Non-Covalent Interactions

**Sodium caseinate + Epicatechin, Binding energy = −10.80 kcal/mol**					
**Hydrophobic Interactions**					
**Residue**	**Amino acid**	**Distance**	**Ligand atom**	**Protein atom**	
54A	GLN	3.70	3411	854	
54A	GLN	3.53	3413	855	
59A	VAL	3.91	3411	934	
60A	TYR	3.70	3413	948	
**Hydrogen Bonds**					
**Residue**	**Amino acid**	**Distance**	**Protein angle**	**Donor atom**	**Acceptor atom**
54A	GLN	3.16	155.61	852 [Nam]	3399 [O3]
56A	GLN	3.19	102.07	883 [Nam]	3401 [O2]
57A	SER	2.80	153.08	3401 [O2]	905 [O2]
60A	TYR	3.57	162.36	946 [Nam]	3398 [O2]
62A	PHE	3.91	123.50	3402 [O2]	991 [O2]

**β-lactoglobulin + Epicatechin, Binding energy = −14.74 kcal/mol**					
**Hydrophobic Interactions**					
**Residue**	**Amino acid**	**Distance**	**Ligand atom**	**Protein atom**	
20A	TYR	3.64	1337	154	
**Hydrogen Bonds**					
**Residue**	**Amino acid**	**Distance**	**Protein angle**	**Donor atom**	**Acceptor atom**
18A	THR	3.60	154.92	131 [O3]	1329 [O2]
43A	VAL	2.97	145.94	1327 [O2]	321 [O2]
59A	GLN	3.63	156.80	451 [Nam]	1326 [O2]
157A	GLU	3.07	118.03	1326 [O2]	1232 [O2]
157A	GLU	3.17	160.13	1325 [O3]	1236 [O3]

Table S5. Quantitative microstructural parameters from SEM image analysis (Origin 2025)

**Parameters**	**F1**	**F2**	**F3**	p-value
Homogeneity index	0.78±0.04	0.65±0.05	0.92±0.03	<0.01
Porosity (% voids)	8.1±0.6	12.4±1.1	4.3±0.4	<0.01
Defect density	1.8±0.3	3.2±0.4	0.7±0.2	<0.01

Means±SD, *n*=50 fields per formulation from triplicate images.

Table S6. DSC thermal transition parameters of MEBs

**Transition**	**F1 (onset/peak/ΔH)**	**F2 (onset/peak/ΔH)**	**F3 (onset/peak/ΔH)**
Tg (°C, J/g)	48/52/15.2	51/56/18.4	46/50/12.8
Tg (°C, J/g)	87/92/8.4	89/94/9.1	85/90/7.2
Maillard (°C)	125/132/6.7	128/136/7.5	122/130/5.9
Total ΔH (J/g)	30.3	35.0	25.9

Triplicate analyses, ±1.2°C / ±0.8 J/g

Table S7. Structure-texture correlations

Parameter	Springiness	Cohesiveness	Hardness	Sensory Score
Homogeneity index	*r* = 0.92*	*r* = 0.88*	*r* = -0.76	*r* = 0.87*
Porosity	*r* = -0.89*	*r* = -0.91*	*r* = 0.87*	*r* = -0.85*
Total ΔH	*r* = -0.87*	*r* = -0.82*	*r* = 0.79 *	*r* = -0.84*

r = correlation coefficient, * = $p<0.01$ (*n* = 9, triplicate data)*
